# (2*E*)-1-(4-Amino­phen­yl)-3-(2,4-dichloro­phen­yl)prop-2-en-1-one

**DOI:** 10.1107/S1600536811020460

**Published:** 2011-06-11

**Authors:** Shailja Singh, Manavendra K. Singh, Alka Agarwal, Firasat Hussain, Satish K. Awasthi

**Affiliations:** a Chemical Biology Laboratory, Department of Chemistry, University of Delhi, Delhi 110 007, India; b Department of Medicinal Chemistry, Institute of Medical Sciences, Banaras Hindu University, Varanasi 225 001, Uttar Pradesh, India; c Nanoscience and Nanotechnology, Department of Chemistry, University of Delhi, Delhi 110 007, India

## Abstract

The title compound, C_15_H_11_Cl_2_NO, is approximately planar (r.m.s. deviation = 0.062 Å) and contains a single C=C double bond in a *trans* (*E*) configuration. The crystal packing is stabilized by intermolecular N—H⋯N and N—H⋯O inter­molecular hydrogen bonding.

## Related literature

For related flavonoids, see: Bargellini & Marini-Bettolo (1940 ▶). For isoflavonoids, see: Nógrádi & Szöllösy (1996 ▶). For the biological activities of chalcones, see: Go *et al.* (2005 ▶); Hans *et al.* (2010 ▶); Trivedi *et al.* (2007 ▶); Nielsen *et al.* (2004 ▶). For anti­malarial activity, see: Mishra *et al.* (2008 ▶). For anti­filarial activity, see: Awasthi, Mishra, Dixit *et al.* (2009 ▶). For other chalcone crystal structures and small mol­ecules, see: Fun *et al.* (2008 ▶); Li *et al.* (2009 ▶); Singh *et al.* (2011 ▶). For the synthesis, see: Migrdichian (1957 ▶); Awasthi, Mishra, Kumar *et al.* (2009 ▶). For inter­molecular N—H⋯N and N—H⋯O hydrogen bonding, see: Fonar *et al.* (2001 ▶).
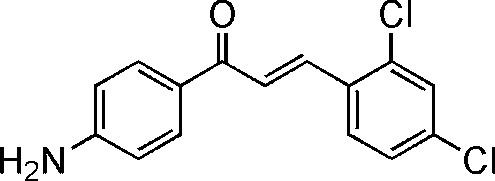


## Experimental

### 

#### Crystal data

C_15_H_11_Cl_2_NO
*M* *_r_* = 292.15
Monoclinic, 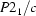

*a* = 22.771 (2) Å
*b* = 3.9889 (5) Å
*c* = 14.7848 (18) Å
β = 92.401 (12)°
*V* = 1341.7 (3) Å^3^
*Z* = 4
Mo *K*α radiation
μ = 0.47 mm^−1^
*T* = 293 K
0.23 × 0.11 × 0.08 mm

#### Data collection

Oxford Diffraction Xcalibur Sapphire3 diffractometer
Absorption correction: multi-scan (*CrysAlis PRO*; Oxford Diffraction, 2009 ▶) *T*_min_ = 0.597, *T*_max_ = 1.000
5765 measured reflections
2625 independent reflections
1733 reflections with *I* > 2σ(*I*)
*R*_int_ = 0.047
Standard reflections: 0

#### Refinement

*R*[*F* ^2^ > 2σ(*F* ^2^)] = 0.057
*wR*(*F* ^2^) = 0.159
*S* = 0.98
2625 reflections
216 parameters
All H-atom parameters refined
Δρ_max_ = 0.33 e Å^−3^
Δρ_min_ = −0.29 e Å^−3^

### 

Data collection: *CrysAlis PRO* (Oxford Diffraction, 2009 ▶); cell refinement: *CrysAlis PRO*; data reduction: *CrysAlis PRO*; program(s) used to solve structure: *SHELXS97* (Sheldrick, 2008 ▶); program(s) used to refine structure: *SHELXL97* (Sheldrick, 2008 ▶); molecular graphics: *Mercury* (Macrae *et al.*, 2006 ▶); software used to prepare material for publication: *publCIF* (Westrip, 2010 ▶).

## Supplementary Material

Crystal structure: contains datablock(s) I, global. DOI: 10.1107/S1600536811020460/zj2011sup1.cif

Structure factors: contains datablock(s) I. DOI: 10.1107/S1600536811020460/zj2011Isup2.hkl

Supplementary material file. DOI: 10.1107/S1600536811020460/zj2011Isup3.cml

Additional supplementary materials:  crystallographic information; 3D view; checkCIF report

## Figures and Tables

**Table 1:** Hydrogen-bond geometry (Å, °)

*D*—H⋯*A*	*D*—H	H⋯*A*	*D*⋯*A*	*D*—H⋯*A*
N1—H1N1⋯O1^i^	0.78 (3)	2.210	2.977 (4)	171 (3)
N1—H2N1⋯N1^ii^	0.76 (4)	2.469	3.134 (5)	147 (4)

Symmetry codes: (i) 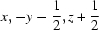; (ii) 
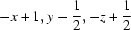
.

**Fig. 1.**
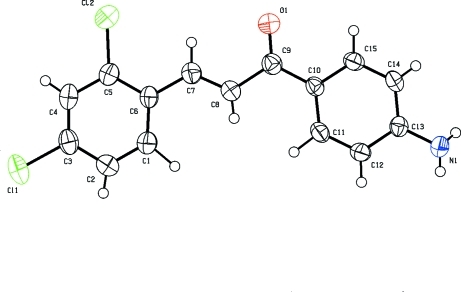
ORTEP diagram of the molecule with thermal ellipsoids drawn at 50% probability level Color code: White: C; red: O; blue: N; white: H; Green: Cl; Green: F

**Fig. 2.**
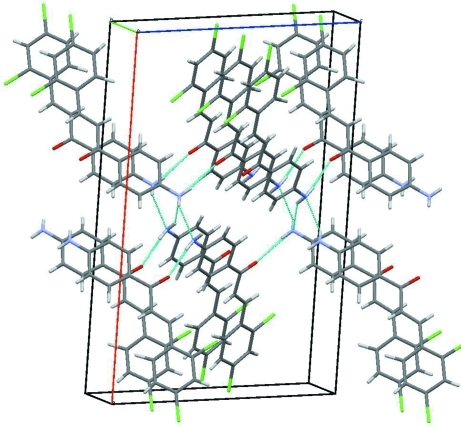
Packing diagram of molecule viewed through b plane showing Intermolecular hydrogen bonding.

**Fig. 3.**
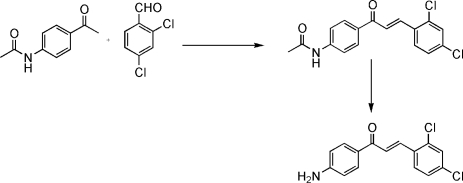
The formation of the title compound.

## supplementary crystallographic information

### Comment

Chalcones (*trans*-1,3-diphenyl-2-propen-1-ones) are precursor of various natural products such as flavonoids (Bargellini & Marini-Bettolo, 1940), isoflavanoids (Nógrádi & Szöllösy, 1996) and key intermediates for synthesis of various heterocyclic scaffolds. Chalcone consists of two aromatic rings joined together by a three carbon α, β-unsaturated carbonyl system (Figure 1). These compounds have broad range of biological activities such as anticancer (Go *et al.*, 2005), antimalarial activity (Mishra *et al.* 2008), anti-TB activity (Hans *et al.* 2010), antiviral (Trivedi *et al.*, 2007), antibacterial (Nielsen *et al.*, 2004) and more recently antifilarial activity (Awasthi, Mishra, Dixit *et al.* 2009) *etc*. Further, SAR on substituted chalcones reveal that presence of α, β-unsaturated ketone is critical for activity in which double bond is in a *trans* (E)-configuration (Li *et al.*, 2009). The crystal structures of few substituted chalcones have been recently reported (Fun *et al.*, 2008; Li *et al.*, 2009). As a part of our ongoing research work on antimicrobial activities of substituted chalcones and crystal structure analysis of small molecules (Singh *et al.*, 2011), we further explored the possibility of characterization of chalcone in the solid state. We crystallized substituted chalcone (2E)-1-(4-aminophenyl)-4-(2,4-dichlorophenyl) but-2-en-1-one,in the mixture of methanol and acetone at room temperature. In this paper, we report the single-crystal X-ray structure of the title compound and possible role of hydrogen bonding in the structure stabilization. The crystal packing is stabilized by intermolecular hydrogen bonding between N1-H2N1···N1 and N1-H1N1···O1(Fonar *et al.*, 2001).as shown in packing diagram along *b* axis(figure 2, table 1).The torsion angle between atom C7—C8 - C9—C10 is 177.8 (3)°. The aminophenyl ring, dichlorophenyl ring and central ketone group are in the same plane, thus molecule is planer. The CCDC No. of the crystal is 797089.

### Experimental

The synthesis of the title compound was carried out according to the published procedure (Migrdichian 1957; Awasthi, Mishra, Kumar *et al.*, 2009). Briefly, an aqueous solution of sodium hydroxide (10%, 10 ml) was added to a solution of acetlylated 4-aminoacetophenone (1.77 g m, 10 mmol) and 2, 4-dichlorobenzaldehyde (1.73 g m, 10 mmol) in minimum amount of methanol (3–5 ml) at ice cooled flask. The reaction mixture was allowed to draw closer to room temperature and stirred for 18–20 hrs yielded a yellow solid. The completion of the reaction was monitored by thin layer chromatography. After completion of the reaction, the mixture was neutralized with 10% hydrochloric acid in water. The acetyl group was removed by refluxing with HCl/C_2_H_5_OH for 4hrs. The product was recrystallized from dry methanol and acetone in 1:1 ratio. After few weeks, light yellow single crystals were obtained. Yield 70%. *R*_f_ = 0.64 (CHCl_3_: MeOH, 99:1). MS (Macromass G) m/*z* = 292.16 (*M*^+^). Elemental analysis (Perkin Elmer): Calcd. for C_15_ H_11_ Cl_2_ NO: C 61.67, H 3.79, Cl 24.26, N 4.79, O 5.48%. Found C 61.70, H 3.81, Cl 24.23, N 4.83, O 5.44%. IR (Perkin Elmer Fourier transform Spectrometer with KBr pellets (cm^-1^): 3462.18–3369.75 (–NH_2_), 2925.42- 2851.54 (aromatic), 1704.23 (C=O in conjugation C=C), 1656.19 (C=C str aromatic), 1584.94 (C=C str in conjugation CO—C=C), 1269.49 (C—N str), 1018.68–1105.40 (C—O—C str), C—Cl (867.02–814.49). ^13^C-NMR (CDCl_3_, 300 MHz): δ 186.66, 152.16, 136.54, 135.27, 135.13, 131.78, 130.75, 130.22, 129.38, 128.07, 127.03, 126.46, 124.75, 113.20, 113.00. ^1^HNMR (Brucker AMX, 300 MHz, CDCl_3_): δ 4. 196 (s, 2H, NH_2_), δ 6.7 (d, 2H, H_2_ and H_6_, J = 8.7 Hz), δ 7.92 (d, 2H, H_3_ and H_5_, J = 8.4 Hz), δ 7.46 (d, 1H, H_α_, J = 15.6 Hz), δ 8.06 (d, 1H, H_β_, J = 15.6 Hz), δ 7.463 (s, 1H, H'_3_), δ 7.29 (d, 1H, H'_5_, J = 6.9 Hz), δ 7.67 (d, 1H, H'_6_, J = 8.4 Hz).

### Refinement

All the H atoms were located from difference Fourier map [range of C—H = 0.81 (4) - 1.10 (3) Å] and N–H = 0.76 (4)–0.78 (4)] and allowed to refine freely.

### Crystal data

**Table:**

C_15_H_11_Cl_2_NO	*F*(000) = 600
*M**_r_* = 292.15	*D*_x_ = 1.446 Mg m^−^^3^
Monoclinic, *P*2_1_/*c*	Mo *K*α radiation, λ = 0.71073 Å
Hall symbol: -P 2ybc	Cell parameters from 1458 reflections
*a* = 22.771 (2) Å	θ = 3.0–29.0°
*b* = 3.9889 (5) Å	µ = 0.47 mm^−^^1^
*c* = 14.7848 (18) Å	*T* = 293 K
β = 92.401 (12)°	Rod, yellow
*V* = 1341.7 (3) Å^3^	0.23 × 0.11 × 0.08 mm
*Z* = 4	

### Data collection

**Table:**

Oxford Diffraction Xcalibur Sapphire3 diffractometer	2625 independent reflections
Radiation source: fine-focus sealed tube	1733 reflections with *I* > 2σ(*I*)
graphite	*R*_int_ = 0.047
ω scans	θ_max_ = 26.0°, θ_min_ = 3.2°
Absorption correction: multi-scan (*CrysAlis PRO*; Oxford Diffraction, 2009)	*h* = −25→28
*T*_min_ = 0.597, *T*_max_ = 1.000	*k* = −3→4
5765 measured reflections	*l* = −17→18

### Refinement

**Table:**

Refinement on *F*^2^	Primary atom site location: structure-invariant direct methods
Least-squares matrix: full	Secondary atom site location: difference Fourier map
*R*[*F*^2^ > 2σ(*F*^2^)] = 0.057	Hydrogen site location: inferred from neighbouring sites
*wR*(*F*^2^) = 0.159	All H-atom parameters refined
*S* = 0.98	*w* = 1/[σ^2^(*F*_o_^2^) + (0.0919*P*)^2^] where *P* = (*F*_o_^2^ + 2*F*_c_^2^)/3
2625 reflections	(Δ/σ)_max_ = 0.003
216 parameters	Δρ_max_ = 0.33 e Å^−^^3^
0 restraints	Δρ_min_ = −0.29 e Å^−^^3^

### Special details

**Table:**

Geometry. All e.s.d.'s (except the e.s.d. in the dihedral angle between two l.s. planes) are estimated using the full covariance matrix. The cell e.s.d.'s are taken into account individually in the estimation of e.s.d.'s in distances, angles and torsion angles; correlations between e.s.d.'s in cell parameters are only used when they are defined by crystal symmetry. An approximate (isotropic) treatment of cell e.s.d.'s is used for estimating e.s.d.'s involving l.s. planes.
Refinement. Refinement of *F*^2^ against ALL reflections. The weighted *R*-factor *wR* and goodness of fit *S* are based on *F*^2^, conventional *R*-factors *R* are based on *F*, with *F* set to zero for negative *F*^2^. The threshold expression of *F*^2^ > σ(*F*^2^) is used only for calculating *R*-factors(gt) *etc*. and is not relevant to the choice of reflections for refinement. *R*-factors based on *F*^2^ are statistically about twice as large as those based on *F*, and *R*- factors based on ALL data will be even larger.

### Fractional atomic coordinates and isotropic or equivalent isotropic displacement parameters (Å^2^)

**Table:**

	*x*	*y*	*z*	*U*_iso_*/*U*_eq_	
C1	0.85377 (14)	0.5099 (8)	0.5485 (2)	0.0466 (8)	
C2	0.91136 (15)	0.6162 (9)	0.5505 (2)	0.0496 (8)	
C3	0.93472 (13)	0.7698 (8)	0.6273 (2)	0.0454 (8)	
C4	0.90165 (14)	0.8197 (8)	0.7010 (2)	0.0465 (8)	
C5	0.84351 (14)	0.7060 (8)	0.6984 (2)	0.0440 (7)	
C6	0.81845 (12)	0.5488 (7)	0.62200 (19)	0.0376 (7)	
C7	0.75725 (13)	0.4285 (8)	0.6200 (2)	0.0434 (8)	
C8	0.72868 (14)	0.2879 (9)	0.5517 (2)	0.0452 (8)	
C9	0.66735 (13)	0.1629 (7)	0.5571 (2)	0.0403 (7)	
C10	0.63905 (12)	−0.0047 (7)	0.47851 (18)	0.0334 (6)	
C11	0.66507 (13)	−0.0353 (8)	0.3948 (2)	0.0408 (7)	
C12	0.63760 (13)	−0.1948 (8)	0.3229 (2)	0.0421 (7)	
C13	0.58122 (12)	−0.3296 (7)	0.33087 (19)	0.0344 (7)	
C14	0.55456 (13)	−0.2959 (8)	0.4125 (2)	0.0384 (7)	
C15	0.58231 (13)	−0.1377 (8)	0.4847 (2)	0.0392 (7)	
N1	0.55305 (14)	−0.4825 (8)	0.2576 (2)	0.0443 (7)	
O1	0.64210 (10)	0.1991 (6)	0.62783 (15)	0.0604 (7)	
Cl1	1.00725 (4)	0.9059 (2)	0.63141 (7)	0.0648 (3)	
Cl2	0.80366 (5)	0.7782 (3)	0.79390 (6)	0.0786 (4)	
H1	0.8413 (15)	0.404 (8)	0.482 (2)	0.057 (9)*	
H1N1	0.5731 (15)	−0.545 (9)	0.220 (2)	0.041 (10)*	
H2	0.9359 (16)	0.602 (9)	0.509 (2)	0.058 (10)*	
H2N1	0.5329 (19)	−0.618 (10)	0.275 (3)	0.067 (15)*	
H4	0.9136 (16)	0.923 (9)	0.749 (3)	0.065 (11)*	
H7	0.7369 (18)	0.430 (10)	0.674 (3)	0.078 (12)*	
H8	0.7462 (17)	0.234 (9)	0.505 (3)	0.065 (12)*	
H11	0.7039 (18)	0.068 (10)	0.384 (3)	0.075 (11)*	
H12	0.6574 (14)	−0.213 (7)	0.271 (2)	0.042 (8)*	
H14	0.5245 (17)	−0.397 (9)	0.422 (2)	0.060 (11)*	
H15	0.5611 (13)	−0.119 (7)	0.537 (2)	0.038 (8)*	

### Atomic displacement parameters (Å^2^)

**Table:**

	*U*^11^	*U*^22^	*U*^33^	*U*^12^	*U*^13^	*U*^23^
C1	0.0408 (17)	0.055 (2)	0.0431 (18)	−0.0017 (16)	−0.0039 (14)	−0.0028 (16)
C2	0.0424 (18)	0.056 (2)	0.051 (2)	−0.0029 (16)	0.0079 (16)	−0.0013 (17)
C3	0.0372 (15)	0.0416 (17)	0.056 (2)	−0.0002 (14)	−0.0094 (15)	0.0068 (15)
C4	0.0461 (18)	0.0424 (19)	0.050 (2)	−0.0059 (15)	−0.0119 (16)	−0.0010 (16)
C5	0.0446 (17)	0.0468 (18)	0.0400 (16)	−0.0010 (15)	−0.0043 (14)	−0.0017 (14)
C6	0.0349 (15)	0.0369 (16)	0.0403 (16)	−0.0002 (13)	−0.0054 (13)	0.0005 (13)
C7	0.0380 (16)	0.0498 (19)	0.0424 (17)	−0.0030 (15)	−0.0007 (14)	−0.0002 (15)
C8	0.0368 (16)	0.057 (2)	0.0421 (18)	−0.0077 (15)	0.0036 (15)	−0.0031 (16)
C9	0.0370 (15)	0.0422 (18)	0.0414 (17)	0.0001 (14)	−0.0004 (14)	0.0033 (14)
C10	0.0268 (13)	0.0373 (16)	0.0361 (15)	0.0007 (12)	−0.0003 (11)	0.0033 (12)
C11	0.0286 (14)	0.0513 (19)	0.0426 (17)	−0.0049 (14)	0.0017 (13)	0.0028 (15)
C12	0.0338 (15)	0.057 (2)	0.0362 (16)	0.0001 (15)	0.0057 (13)	−0.0007 (15)
C13	0.0303 (14)	0.0349 (16)	0.0374 (15)	0.0042 (12)	−0.0043 (12)	0.0009 (12)
C14	0.0275 (15)	0.0425 (18)	0.0452 (17)	−0.0054 (14)	0.0007 (13)	0.0031 (14)
C15	0.0333 (15)	0.0470 (19)	0.0378 (16)	0.0013 (14)	0.0068 (13)	0.0016 (14)
N1	0.0372 (15)	0.054 (2)	0.0409 (16)	−0.0038 (15)	−0.0023 (13)	−0.0097 (15)
O1	0.0515 (13)	0.0868 (19)	0.0436 (13)	−0.0177 (13)	0.0096 (11)	−0.0133 (12)
Cl1	0.0387 (5)	0.0691 (6)	0.0857 (7)	−0.0114 (4)	−0.0079 (4)	0.0039 (5)
Cl2	0.0705 (6)	0.1094 (9)	0.0565 (6)	−0.0218 (6)	0.0104 (5)	−0.0299 (6)

### Geometric parameters (Å, °)

**Table:**

C1—C2	1.377 (5)	C9—O1	1.223 (4)
C1—C6	1.388 (4)	C9—C10	1.466 (4)
C1—H1	1.10 (3)	C10—C11	1.399 (4)
C2—C3	1.377 (5)	C10—C15	1.403 (4)
C2—H2	0.85 (4)	C11—C12	1.368 (4)
C3—C4	1.365 (5)	C11—H11	0.99 (4)
C3—Cl1	1.737 (3)	C12—C13	1.401 (4)
C4—C5	1.399 (5)	C12—H12	0.91 (3)
C4—H4	0.86 (4)	C13—C14	1.381 (4)
C5—C6	1.393 (4)	C13—N1	1.378 (4)
C5—Cl2	1.734 (3)	C14—C15	1.371 (4)
C6—C7	1.473 (4)	C14—H14	0.81 (4)
C7—C8	1.305 (4)	C15—H15	0.93 (3)
C7—H7	0.94 (4)	N1—H1N1	0.78 (4)
C8—C9	1.488 (4)	N1—H2N1	0.76 (4)
C8—H8	0.84 (4)		
			
C2—C1—C6	122.1 (3)	O1—C9—C10	121.7 (3)
C2—C1—H1	110.2 (18)	O1—C9—C8	118.8 (3)
C6—C1—H1	127.6 (18)	C10—C9—C8	119.5 (3)
C3—C2—C1	119.3 (3)	C11—C10—C15	116.7 (3)
C3—C2—H2	113 (2)	C11—C10—C9	123.5 (3)
C1—C2—H2	128 (2)	C15—C10—C9	119.7 (3)
C4—C3—C2	121.1 (3)	C12—C11—C10	122.1 (3)
C4—C3—Cl1	118.8 (2)	C12—C11—H11	117 (2)
C2—C3—Cl1	120.1 (3)	C10—C11—H11	121 (2)
C3—C4—C5	119.0 (3)	C11—C12—C13	120.2 (3)
C3—C4—H4	125 (3)	C11—C12—H12	117.7 (19)
C5—C4—H4	116 (3)	C13—C12—H12	122.0 (19)
C6—C5—C4	121.5 (3)	C14—C13—N1	121.6 (3)
C6—C5—Cl2	121.6 (2)	C14—C13—C12	118.3 (3)
C4—C5—Cl2	116.8 (2)	N1—C13—C12	120.1 (3)
C1—C6—C5	117.0 (3)	C15—C14—C13	121.4 (3)
C1—C6—C7	121.8 (3)	C15—C14—H14	117 (3)
C5—C6—C7	121.2 (3)	C13—C14—H14	120 (3)
C8—C7—C6	126.6 (3)	C14—C15—C10	121.2 (3)
C8—C7—H7	114 (2)	C14—C15—H15	116.4 (18)
C6—C7—H7	119 (2)	C10—C15—H15	122.4 (18)
C7—C8—C9	122.8 (3)	C13—N1—H1N1	116 (2)
C7—C8—H8	120 (3)	C13—N1—H2N1	109 (3)
C9—C8—H8	116 (3)	H1N1—N1—H2N1	113 (4)
			
C6—C1—C2—C3	−0.8 (5)	C7—C8—C9—O1	1.5 (5)
C1—C2—C3—C4	−0.4 (5)	C7—C8—C9—C10	−177.5 (3)
C1—C2—C3—Cl1	180.0 (2)	O1—C9—C10—C11	176.4 (3)
C2—C3—C4—C5	1.3 (5)	C8—C9—C10—C11	−4.6 (4)
Cl1—C3—C4—C5	−179.1 (2)	O1—C9—C10—C15	−2.1 (4)
C3—C4—C5—C6	−1.2 (5)	C8—C9—C10—C15	176.8 (3)
C3—C4—C5—Cl2	−179.8 (2)	C15—C10—C11—C12	−1.8 (4)
C2—C1—C6—C5	0.9 (5)	C9—C10—C11—C12	179.7 (3)
C2—C1—C6—C7	−178.8 (3)	C10—C11—C12—C13	1.0 (5)
C4—C5—C6—C1	0.1 (5)	C11—C12—C13—C14	0.2 (4)
Cl2—C5—C6—C1	178.6 (2)	C11—C12—C13—N1	178.3 (3)
C4—C5—C6—C7	179.7 (3)	N1—C13—C14—C15	−178.7 (3)
Cl2—C5—C6—C7	−1.7 (4)	C12—C13—C14—C15	−0.6 (4)
C1—C6—C7—C8	−2.6 (5)	C13—C14—C15—C10	−0.2 (5)
C5—C6—C7—C8	177.8 (3)	C11—C10—C15—C14	1.4 (4)
C6—C7—C8—C9	177.8 (3)	C9—C10—C15—C14	180.0 (3)

### Hydrogen-bond geometry (Å, °)

**Table:**

*D*—H···*A*	*D*—H	H···*A*	*D*···*A*	*D*—H···*A*
N1—H1N1···O1^i^	0.78 (3)	2.210	2.977 (4)	171 (3)
N1—H2N1···N1^ii^	0.76 (4)	2.469	3.134 (5)	147 (4)

Symmetry codes: (i) *x*, −*y*−1/2, *z*+1/2; (ii) −*x*+1, *y*−1/2, −*z*+1/2.
